# Psychiatric Comorbidities in Children and Adolescents with High-Functioning Autism Spectrum Disorder: A Study on Prevalence, Distribution and Clinical Features in an Italian Sample

**DOI:** 10.3390/jcm12020677

**Published:** 2023-01-14

**Authors:** Elisa Fucà, Silvia Guerrera, Giovanni Valeri, Laura Casula, Roberta Lucia Novello, Deny Menghini, Stefano Vicari

**Affiliations:** 1 Child and Adolescent Neuropsychiatry Unit, Bambino Gesù Children’s Hospital (IRCCS), 00146 Rome, Italy; 2 Department of Life Science and Public Health, Università Cattolica del Sacro Cuore, 00168 Rome, Italy

**Keywords:** adaptive skills, behavioral problems, emotional problems, gender, neurodevelopmental disorders, psychiatric disorders

## Abstract

This study investigated the prevalence and distribution of psychiatric comorbidities in a group of 472 children and adolescents with ASD aged 3–18 years. We examined differences in age, sex, IQ, adaptive skills, and ASD symptom severity by comparing participants with ASD (ASD group) with participants with ASD and a psychiatric disorder (ASD/PSY group). Overall, 32.2% of participants had a comorbid psychiatric condition. Attention deficit/hyperactivity disorder (ADHD) was the most frequent diagnosis among preschoolers (20.4%); among school-age children, ADHD and anxiety/obsessive-compulsive disorders were the most frequent conditions (21% and 10.6%, respectively); finally, adolescents exhibit higher prevalence of anxiety/obsessive-compulsive disorders (21.8%). The ASD/PSY group showed a higher percentage of males, they were older and showed lower adaptive skills than the group with ASD; moreover, their mothers exhibited higher stress levels than mothers of participants in the ASD group. The comparison between age groups in participants within ASD/PSY group revealed that preschoolers had lower IQ than school-age children and adolescents, and worse adaptive skills, more repetitive behaviors, and restricted interests than adolescents. This study highlights the importance of an accurate diagnosis of psychiatric comorbidities in children and adolescents with ASD, also considering individual and family impairment.

## 1. Introduction

Autism Spectrum Disorder (ASD) is a neurodevelopmental disorder characterized by deficits in social communication and restricted interests and repetitive behaviors [1]. The etiology is multifactorial and genetic factors play a principal role. ASD is increased in siblings and rare genetic variants appear to be causally linked with ASD, including copy number variations of several genes; to date, more than 100 genes and genomic regions have now been linked with ASD [2]. A recent systematic review reported a median prevalence of 65/10,000, with increase in measured prevalence over time either at a country level, such as the United States, South Korea, and France [3]. The reported male–female ratio is of 3:1 [4].

In addition to impairment in sociolinguistic communication and behavioral skills, abnormal sensory processing, in terms of hyper- or hypo-reactivity to sensory input, has been included in the DSM-5 diagnostic criteria for ASD. The prevalence of atypical responses to sensory stimuli ranges from 60 to 96% in children with ASD and appears to be related not only with cognitive and social impairment [5] but also with comorbid conditions, such as gastrointestinal and sleep problems [6,7]. Individuals with ASD are significantly more likely to develop a variety of comorbid medical and psychiatric conditions than typically developing children [8]. For example, language delays, motor problems, seizures, sleep disturbances, eating difficulties (such as food selectivity), gastrointestinal problems, and high levels of activity are frequently reported in preschoolers [9,10,11]. In addition, the proportion of individuals with ASD exhibiting co-occurring intellectual disability has been estimated at 33% [3]. When medical, psychiatric, and developmental disorders are associated with ASD, it is necessary to provide an accurate and individualized clinical assessment, taking into account specific symptoms, prognosis, and treatment indications.

With the new classification of neurodevelopmental disorders proposed by the DSM-5, which includes overcoming the exclusion criteria proposed by the DSM-IV-Text Revision and the recognition of multiple associated symptoms in complex clinical pictures, we currently have the possibility of identifying comorbid psychiatric disorders in children with ASD. This has probably contributed to the increased research interest in the topic. In fact, to date, many systematic reviews, meta-analyses, and umbrella reviews summarize research findings on this topic. According to the overall pooled prevalence estimates of mental health conditions in ASD reported by Lai and collaborators [12], 28% are attention deficit/hyperactivity disorder (ADHD), 20% are anxiety disorders; 13% are sleep–wake disorders; 12% are disruptive, impulse-control, and conduct disorders; 11% are depressive disorders; 9% are obsessive compulsive disorder (OCD); 5% are bipolar disorders; and 4% are schizophrenia spectrum disorders. A more recent umbrella review, which included 14 systematic reviews and 12 meta-analyses, revealed a variable prevalence index of psychiatric comorbidities in ASD, ranging from 54.8% up to 94%, among the participants considered [13]. The authors reported the following prevalence ranges among the studies: anxiety disorders ranged between 1.47% and 54%; depressive disorder ranged from 2.5% to 47.1%, bipolar disorders ranged from 6% to 21.4%; schizophrenia spectrum and other psychotic disorders ranged from 4% to 67%; suicidal ideation and attempt ranged from 1% to 66%; OCD ranged from 9% to 22%; disruptive, impulse-control, and conduct disorders ranged from 12% to 48%; finally, ADHD ranged from 25.7% to 65%.

In summary, considerable heterogeneity emerges in prevalence estimates of comorbid psychiatric conditions in ASD. Prevalence estimates vary according to the context of the sample (e.g., from psychiatry referrals or schools), the methodology used to assess the presence of psychiatric conditions (parent-report, self-report or assessed), the age of the participants and the level of their cognitive abilities [2].

In particular, the age of participants is a crucial variable in understanding the developmental trajectories and mental health outcomes of individuals with ASD. Several studies that have focused on prevalence rates of psychiatric conditions in young individuals with ASD have shown an increase in psychiatric conditions with age, resulting in a greater need for treatment and care [12,14,15,16,17,18,19]. However, a recent systematic review revealed a substantial heterogeneity even among studies focusing on the prevalence of comorbidities in youth with ASD [20]. Indeed, the authors reported wide prevalence ranges for each psychiatric comorbidity, namely: 0–86% for ADHD, 0.0–82.2% for anxiety, 0.0–38.6% for depressive disorders. 

Geographic differences could also help explain this wide variability among studies. Extending to the field of psychiatric comorbidities some previous considerations on ASD prevalence estimates, the potential impact of geographical and socioeconomic factors on prevalence rates of psychiatric comorbidities in ASD cannot be excluded [21]. Therefore, research estimating prevalence in different areas is highly required. As concerns Italy, studies are available on the prevalence of psychiatric comorbidities in both preschoolers [22] and school-aged children and adolescents [23], whereas other research focused on specific diagnoses, such as schizophrenia [24].

Sex is another important variable to consider in characterizing psychiatric comorbidities in individuals with ASD. Literature has provided inconsistent results, with some studies reporting no sex differences in the distribution of psychiatric comorbidities [25,26,27], others reporting a higher prevalence of emotional and behavioral problems in females [28], and still others showing a higher prevalence of both externalizing and internalizing symptoms in males [19,26,27]. 

Accumulating evidence indicates the presence of comorbid psychiatric disorders, including the co-occurrence of other neurodevelopmental disorders, such as ADHD, might not only hamper prompt recognition of ASD, but also exacerbate ASD symptoms themselves [29], interfering with prognosis and ultimately requiring specific intervention approaches [30]. In addition, comorbid psychiatric disorders in ASD are associated with increased use of psychotropic medication and overall healthcare utilization, as well as interruption of ASD-related interventions [31,32,33]. In summary, comorbid psychiatric disorders in ASD increase the likelihood of worse long-term outcomes, including higher mortality risk and impaired quality of life [8,33,34]. 

Considering the impact of comorbid psychiatric conditions on developmental outcomes in ASD, boosting our knowledge about the distribution of different psychiatric disorders in the ASD population represents a primary need to guarantee prompt and adequate interventions for youth with ASD and their families.

Previous reviews and meta-analyses provided extensive and detailed evidence of the high epidemiological burden of psychiatric disorders associated with ASD by examining studies with well-defined methodological criteria, such as those related to ASD diagnosis. The studies conducted to date have also had the merit of highlighting the important role played by psychiatric comorbidity on the functioning of individuals with ASD. However, despite the increased research interest in studying the prevalence and distribution of comorbid psychiatric disorders in ASD, some knowledge gaps persist. First, most of the studies are of English-speaking origin: as pointed out elsewhere, the wide differences on the prevalence and distribution of psychiatric comorbidities in ASD could be due, at least in part, to the heterogeneity in the country of origin of published studies [12]. Therefore, research from different countries around the world is highly desirable [13]. In particular, few studies have reported the prevalence of comorbid psychiatric disorders in youth with ASD from Southern Europe. Second, little evidence is available about the impact of comorbid psychiatric conditions on adaptive skills in individuals with high-functioning ASD. Third, it has been suggested that untangling the heterogeneity of current findings on psychiatric comorbidities in ASD requires more detailed empirical research that takes into account individual and clinical features [12], such as age.

Given these limitations, it seems crucial to conduct more research to understand the burden of psychiatric comorbidities in ASD among different countries, taking into account specific features and the role they played in individual and family impact.

Therefore, the current study had the following three aims:

- Explore the prevalence and distribution of comorbid psychiatric disorders across different age groups in a large group of Italian children and adolescents with high-functioning ASD.
- Investigating the individual and clinical features associated with psychiatric comorbidities, namely age, sex, adaptive skills, ASD symptoms, and maternal stress levels.
- Explore differences among age groups in individual and clinical features associated with psychiatric comorbidities in ASD.

A better understanding of the features associated with psychiatric comorbidities in children and adolescents with ASD is a crucial step towards appropriate clinical processes.

## 2. Materials and Methods

### 2.1. Procedure

The current study employed a cross-sectional design. Data were retrospectively collected from an in-depth review of the files of patients who referred to the Child and Adolescent Neuropsychiatry Unit of a third level Children’s Hospital between 2017 and 2019 for a neuropsychiatric evaluation following pediatrician’s clinical suspicion of ASD or for clinical follow-ups after receiving ASD diagnosis. Routine assessment procedure always included neuropsychiatric examination, cognitive and adaptive functioning evaluation, assessment of ASD symptoms and an accurate psychopathological investigation. Exclusion criteria were as follows: presence of neurological conditions (e.g., epilepsy); presence of genetic disorders; IQ < 70. Since the COVID-19 pandemic may have contributed to the onset of psychopathological symptomatology possibly related to the pandemic itself, we decided to exclude patients who visited from 2020. The study was conducted according to the guidelines of the Declaration of Helsinki and approved by the local Ethics Committee (protocol code: 2423_OPBG_2021, approved on 27 October 2021).

### 2.2. Participants

**Figure 1:**
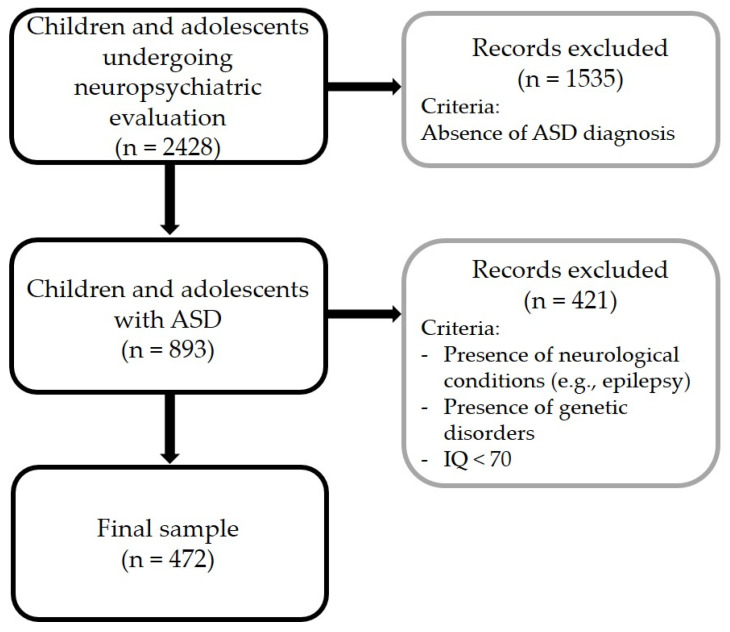
Workflow of the study.

Of 2428 children and adolescents undergoing neuropsychiatric evaluation, 893 children and adolescents were diagnosed with ASD or had a previously established diagnosis of ASD. This initial sample included 737 males and 156 females (82.5% and 17.5% out of 893, respectively). The chronological age of participants ranged from 3 to 18 years (mean 7.3 ± 3.4; mean nonverbal IQ: 79.3 ± 21.9). After the application of the exclusion criteria, the final sample included 472 children and adolescents with ASD (mean age: 7.16 ± 3.4; mean nonverbal IQ: 90.6 ± 19.3). The workflow of the study is summarized in Figure 1.

**Figure 2:**
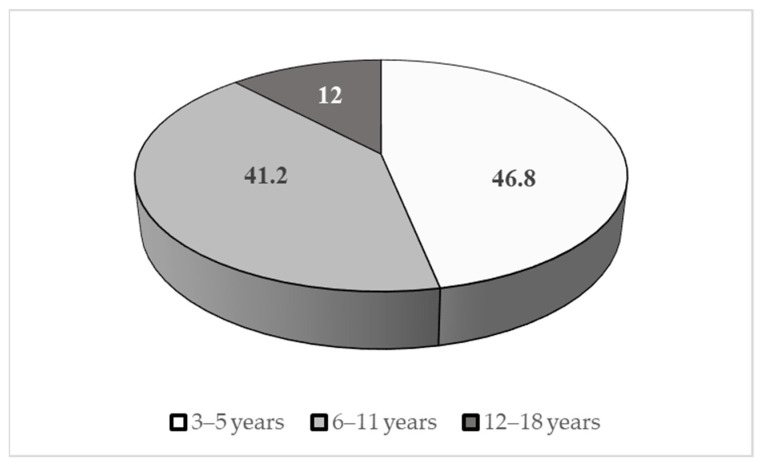
Age distribution of the sample (%).

As concerns sex distribution, 82% were males and 18% females. Age distribution is summarized in Figure 2.

### 2.3. Measures

#### 2.3.1. Autistic Symptoms Assessment

The patients underwent an extensive examination by a team of neuropsychiatrists and psychologists with specific expertise in assessing ASD. The diagnosis of ASD was established in accordance with the DSM-5 and was confirmed by the administration of the “gold-standard” instruments for the assessment of ASD symptoms, namely the Autism Diagnostic Observation Schedule, 2nd Edition (ADOS-2) [34] and the Autism Diagnostic Interview-Revised (ADI-R) [35]. The ADOS-2 is a semi-structured direct assessment of communication, social interaction, and play or imaginative use of materials for individuals with a suspected diagnosis of ASD. The ADOS-2 consists of five modules designed for children and adults with different levels of language, from nonverbal to verbally fluent; it was administered and scored by licensed clinicians. Total score combines symptoms from the Social Affect and Restricted and Repetitive Behaviors domains. In the analyses, raw total scores and comparison scores were considered for the ADOS-2 calibrated severity score (CSS). The instrument has high interrater and test–retest reliability, good predictive value, and good specificity in distinguishing between ASD versus non-spectrum (Module 3 sensitivity of 0.91, specificity of 0.84) [36,37] and it has been adapted for different countries. The ADI-R is a standardized, semi-structured interview during which caregivers report information about an individual suspected of having an ASD. It generates algorithm scores for each of the three subdomains of autistic symptoms: qualitative impairments in reciprocal social behavior; qualitative abnormalities in communication; and restricted range of interests and/or stereotypic behaviors. This interview is appropriate for adults and children with a mental age of 18 months and above, and it takes two hours or longer to administer and score [35,36]. Literature reports sensitivity and specificity values for ASD versus non-ASD ranging from 0.19 to 0.75 for sensitivity and from 0.63 to 1.00 for specificity [38].

#### 2.3.2. Psychopathological Assessment

All participants included in the study underwent a neuropsychiatric examination, including clinical interviews and direct observations, by a team of neuropsychiatrists and clinical psychologists in order to investigate the presence of psychopathological disorders. Clinical interviews were used to investigate the presence of psychopathological disorders according to DSM-5 criteria; clinicians with specific expertise on ASD and developmental psychopathology assessment conducted the interviews. Whenever possible, not only the parents but also the child/adolescent were considered as sources of information. If general symptoms of a psychopathological disorder emerged, detailed questions were used to verify the diagnosis.

#### 2.3.3. Cognitive Assessment

Cognitive development was assessed by Wechsler Intelligence Scale for Children (WISC-IV) [39]. The instrument is made of e 10 core subtests, namely Block Design, Similarities, Digit Span, Picture Concepts, Coding, Vocabulary, Letter–Number Sequencing, Matrix Reasoning, Comprehension and Symbol Search. WISC-IV administration provides four different indexes: Verbal Comprehension Index, Perceptual Reasoning Index, Working Memory Index, and Processing Speed Index.

In cases of language problems, we administered non-verbal instruments. In particular, we used the Leiter International Performance Scale–3rd Edition–Leiter-3 [40]—which provides a nonverbal measure of intelligence and assesses the ability to reason by analogy, by matching and perceptual reasoning in general, irrespective of language and formal schooling. The Global Non-Verbal Intelligent Quotient obtained through this test is based on four subtests: Figure Ground, Form Completion, Classification and Analogies, and Sequential Order. We used also the Colored Progressive Matrices—CPM [41], a 60-item test to assess mental ability associated with abstract reasoning, and considered a nonverbal estimate of fluid intelligence. The test consists of increasingly difficult pattern matching tasks and has little dependency on language abilities. Moreover, we administered the Griffiths Mental Development Scales—Extended Revised 0–2–GMDS-ER 2–8 [42] when children failed to complete the WISC-IV, the Leiter-3 or the CPM for their reduced attentional resources. The GMDS-ER provides a measure of development in children aged 0–2 years in five different domains (Locomotor, Personal–Social, Language, Eye and Hand Coordination, and Performance). Every subscale provides a different developmental quotient and a diagnostic indication of problems in early childhood. The average of the quotients of the six subscales provides a Global Developmental Quotient. In the present study, we considered only the nonverbal scores obtained from each instrument, as follows: the Global Nonverbal Intelligence Quotient (nvIQ) of the Leiter-3, the Performance Scale Quotient of the GMDSER 2–8, the Perceptual Reasoning Index of the WISC-IV, or the IQ of the CPM.

#### 2.3.4. Adaptive Functioning Assessment

Adaptive functioning was assessed using the Vineland Adaptive Behavior scales–Second Edition (VABS II) [43] or the Italian version of Adaptive Behavior Assessment System-Second Edition Parent Form 0–5 and Form 5–21 (ABAS II) [44]. The VABS-II assesses adaptive functioning of individuals from birth to 90 years and 11 months through caregivers’ interviews, and yields three domain scores: Communication, Socialization and Daily Living Skills (the fourth Motor Skills domain is investigated only for children younger than 7 years). An overall Adaptive Behavior Composite score is also provided. The VABS II provides standard scores (M = 100, SD = 15), and higher scores indicate better functioning. Inter-rater reliability is 0.74. The ABAS-II 0–5 and 5–21 is a parent-report questionnaire for caregivers of individuals aged from 0–5 and 5–21 years. ABAS-II yields three specific domain scores (Conceptual, Social, and Practical) and an overall General Adaptive Composite (GAC). In the present study, the GAC Global Adaptive Functioning score of the ABAS II test and the Composite Adaptive Behavior score of the VABS II was considered for the analyses (“Adaptive functioning score”). Test–retest reliability coefficients of the GAC are all in the 0.90s; the inter-rater reliability coefficients on the GAC scores are 0.83–0.85. Both instruments have been widely employed in ASD research [45,46,47].

#### 2.3.5. Maternal Stress Assessment

To investigate maternal stress levels, the Parenting Stress Index-Short Form (PSI) [48] was administered to a subgroup of 402 mothers. PSI is an easy-to administer tool to measure maternal stress. It consists of 36 questions and each item is rated on a 5-point Likert scale from (1) strongly disagree to (5) strongly agree. The PSI captures three domains—parental distress, parent–child dysfunctional interaction, and difficult child. The sum of all questions results in the Total Stress score. Inter-rater correlations for the Total score are 0.52–0.96 [49]. PSI has been translated into several languages and has been frequently used in ASD research [50,51,52,53].

## 3. Results

### 3.1. Prevalence of Psychiatric Comorbidities

Of 472 children and adolescents with ASD, 153 (32.2%) received a diagnosis of a comorbid psychiatric disorder, as follows: ADHD (58.2%); anxiety/OCD, including phobias, generalized and social anxiety disorders (21.6%); mood disorders, including depression and bipolar disorders (5.2%); Oppositional Defiant Disorder (ODD, 5.2%); Tourette’s Disorder/Tic Disorder (4.6%); feeding and eating disorders, including anorexia and avoidant/restrictive food intake disorder (3.3%); other disorders (e.g., psychotic disorders and post-traumatic stress disorder, 1.9%). To better characterize the distribution of psychiatric comorbidities in different age groups, we distinguished between preschoolers (3–5 years), school-age children (6–11 years) and adolescents (12–18 years). In total, 46 out of 221 preschoolers (20.8%) received a diagnosis of psychiatric comorbidity; 82 out of 196 (41.8%) school-age children exhibited a psychiatric comorbidity; finally, 25 out of 55 adolescents (45.5%) presented a co-occurring mental health condition. Distribution of single psychiatric comorbidities is reported in Table 1.

### 3.2. Differences between Groups: Age, Sex, Adaptive Skills, ASD Symptoms and Maternal Stress

The sample was divided into two subgroups:

1. Without psychiatric comorbidities (ASD group; *n* = 319; 79.9/21% males/females);
2. With psychiatric comorbidity (ASD/PSY group; *n* = 153; 87.6/12.4% males/females).

As expected, the two groups did not differ in nvIQ (ASD group: 91.2 ± 19.1; ASD/PSY group: 89 ± 19.8; *p* > 0.05). Chi-square test detected significant differences in sex distribution between groups (X^2^ = 4.2, *p* = 0.041). Means and standard deviations for age, adaptive skills as measured by the ABAS II composite score, ADOS-2 scores and PSI are reported in Table 2. Significant differences between ASD and ASD/PSY groups emerged for age, adaptive skills, and maternal stress.

### 3.3. ASD/PSY Group: Differences between Preschoolers, School-Aged Children and Adolescents

With the aim to explore the individual and clinical features of youth with ASD across different ages, the three age groups (preschoolers, school-aged children, and adolescents) were compared on IQ, adaptive skills, ADOS-2 scores, and PSI scores. ANOVA analysis on IQ, with age group (i.e., preschoolers, school-aged children, and adolescents) as between factor and nvIQ (in years) as within factor showed a significant effect, F (2133) = 6.11, *p* = 0.003, ηp^2^ = 0.95. Post-hoc analyses (Tukey HSD test) showed that preschoolers exhibited significantly lower IQ than school-age children (80.82 ± 19.79 and 91.46 ± 16.76; *p* = 0.011) and adolescents (96.54 ± 21.54; *p* = 0.005). No differences emerged between school-aged children and adolescents. ANOVA analysis on adaptive skills, with age group as the between factor and adaptive functioning score as the within factor, showed a significant effect, F (2133) = 3.551, *p* = 0.031, ηp^2^ = 0.93. Post-hoc analyses (Tukey HSD test) revealed that preschoolers had significantly worse adaptive skills than adolescents (58.24 ± 11.82 and 67.77 ± 18.46, respectively, *p* = 0.043). ANOVA with age group as the between factor and ADOS-CSS scores as within factors showed significant differences between groups, F (2136) = 5.39, *p* = 0.005, ηp^2^ = 0.95. As concerns the Social Affect domain, post-hoc analyses (Tukey HSD test) did not detect significant differences between groups (all *p* > 0.05). As concerns the Repetitive Behaviors domain, post-hoc analyses (Tukey HSD test) revealed that preschoolers exhibited significantly higher scores than adolescents (7.28 ± 1.14 and 5.47 ± 2.87, respectively; *p* = 0.004); no other differences between age groups emerged. As concerns ADOS-2 Total Score, no differences emerged, F (2136) = 1.915, *p* = 0.151, ηp^2^ = 0.93. Finally, ANOVA analysis on differences on maternal stress, with age group as between factor and the PSI Total score as within factor showed no significant effect, F (2119) = 1.09, *p* = 0.34, ηp^2^ = 0.91.

## 4. Discussion

The first aim of this study was to estimate the prevalence and distribution of psychiatric disorders in a group of 472 Italian children and adolescents with a diagnosis of ASD. Of these, 32.2% were diagnosed with psychiatric comorbidity. The highest prevalence of ADHD (58.2%) was observed among youths who received a comorbid psychiatric disorder diagnosis. Second, individual and clinical characteristics, i.e., age, sex, IQ, adaptive skills and ASD symptoms in participants with high-functioning ASD and in participants with ASD diagnosed with a comorbid psychiatric disorder were studied. We found a higher proportion of males in the ASD/PSY group than in the ASD group. In addition, participants in the ASD/PSY group were older and showed lower adaptive skills than the group with ASD; finally, their mothers showed significantly higher levels of stress than mothers of children with ASD alone. Third, analysis of individual and clinical features across age groups in youths diagnosed with comorbid psychiatric disorders revealed that preschoolers had lower IQ, worse adaptive skills and more repetitive behaviors and restricted interests than adolescents.

As suggested by similar previous research, prevalence estimates of psychiatric comorbidity in ASD are very variable [11,18,54,55,56]. A systematic review, including 96 studies conducted between 1993 and 2019 [12], found a wide range (from 4% to 28%) of prevalence estimates of psychiatric conditions associated with ASD, according to the ICD-10, DSM-IV, and DSM-5 classifications. Specifically, the authors identified the highest prevalence of ADHD and anxiety disorder followed by sleep–wake disorders, disruptive impulse control and conduct disorders, depressive disorders, and OCD. In 2020, Hossain and colleagues analyzed 14 systematic reviews and 12 meta-analyses to assess the prevalence of comorbid psychiatric disorders among people with ASD, without age or geographical restriction [13]. Although the percentage of comorbid disorders reported was higher than that found in our study, with at least one psychiatric disorder in comorbidity ranging from 54.8% to 94%, similar to our results a higher prevalence of externalizing disorders was found, followed by anxiety and OCD, mood disorder, and other psychiatric disorders with a very wide prevalence range. As suggested earlier [12,13], differences among studies of psychopathological prevalence estimates in individuals with ASD could be related to multiple factors, such as classification systems, assessment instruments, and demographic characteristics of the samples (mean age, sex distribution) [12]. In particular, differences among population subgroups seem to play a major role. In addition, the socioeconomic conditions and the cultural context could differ among the groups considered for the studies [57,58], influencing prevalence estimates. Most reviews found studies involving youth and adults without any stratification by age. Few studies have focused on psychiatric comorbidities arising exclusively in children and adolescents with ASD [13]. In the present study, we explored the prevalence and distribution of comorbid psychiatric conditions in ASD and specifically on the developmental age. We investigated a large group of children and adolescents with a diagnosis of ASD, according to the DSM-5 classification, and did not rely exclusively, as in other studies, on questionnaire scores, or clinical interview results [27,55]. On the other hand, a focus on the developmental age may have left out a number of subthreshold conditions, such as depression, that gain greater visibility in adults with ASD [59,60]. ADHD appears to be the most common psychiatric comorbidity in preschoolers, and this is in line with previous findings indicating high levels of co-occurrence of ASD and ADHD even in preschoolers [61,62]. With the transition to school age, ADHD remained the most frequently observed comorbidity; however, we observed a substantial improvement of anxiety/OCD diagnoses, which became the most represented psychiatric comorbidities in adolescence, followed by mood disorders and ADHD. These findings are consistent with the work of Lai and collaborators who found a higher prevalence of externalizing disorders in younger children than in older children, and a greater presence of mood and anxiety disorders with increasing age [12].

These results underline how, when assessing psychiatric comorbidities in children with ASD, special attention should be paid to the school-age period, which is critical for the study of psychiatric symptoms. Indeed, most psychiatric conditions might emerge when the child undergoes not only physiological and neurodevelopmental changes (e.g., puberty), but he/she is also exposed to several new environmental stimuli, such as interactions with caregivers, peers, and teachers. This is further supported by another finding from the present research, which indicates that the participants belonging to the ASD/PSY group were older than the youth in the ASD group. Consistently, several studies have shown that comorbid psychiatric conditions in ASD are influenced by age [7,27,53,54]. For example, results from a cross-sectional study indicated that increasing age is the most common risk factor for the development of anxiety and mood disorders in children and adolescents with ASD aged 6–17, regardless of the presence or absence of ADHD [14]. Anxiety disorders can exacerbate ASD symptoms and behavioral problems, causing persistent distress [63,64]. It should be noted that children and adolescents with ASD may present with anxiety with both typical and unconventional manifestations, such as fear of novelty, and/or unusual phobias [65,66]. This clinical heterogeneity, associated with possible overlaps between anxiety manifestation and ASD symptoms, may hamper the assessment of anxiety symptoms in youth with ASD. In addition, knowledge about treatments targeting anxiety in youth with ASD is limited. Data from the Autism Speaks Autism Treatment Network showed modest evidence of the effectiveness of cognitive–behavioral therapy and a lack of randomized placebo-controlled trials investigating pharmacologic interventions for anxiety in youth with ASD [67,68]. However, pilot studies investigating the effectiveness of new intervention strategies for anxiety in youth with ASD have been recently performed [69,70].

The heterogeneity of results regarding the prevalence of psychiatric comorbidities in ASD leads to considerations regarding how comorbidities are assessed in this population. Rosen and colleagues [71] highlighted the pressing need for development and validation of assessment tools specifically developed to capture ASD-specific presentations of co-occurring psychiatric conditions, ascertaining whether these clinical manifestations reflect true, co-occurring conditions or are epiphenomenon of ASD symptomatology. The authors also highlighted the usefulness of assessment tools suitable for individuals with varying verbal and intellectual abilities.

The second aim of the current study was to explore group differences between ASD and ASD/PSY in terms of individual and clinical features. We found that the male-to-female ratio was different between the ASD and ASD/PSY groups, with a significantly higher representation of males in the ASD/PSY group than in the ASD group. This result is consistent with previous findings indicating that psychiatric comorbidities are more common in males than in females with ASD. For example, the study by Brookman-Frazee and colleagues [54] reported higher prevalence of ADHD in boys than in girls, consistent with previous reports [72]. More recent research has reported that males with ASD exhibit more internalizing problems than females in preschool years [22]. However, other studies have found little or no differences in the distribution of psychopathological comorbidities between males and females with ASD [20,22,24,55]. Finally, some authors have reported that females with ASD are significantly more likely than males to have co-occurring emotional and behavioral problems associated with psychopathology in childhood [73,74]. In sum, further research is still needed, taking into account the possible bias related to the presence of comorbidities—such as ADHD—that are often unrecognized and therefore underestimated in females [23]. The comparison between ASD and ASD/PSY groups also detected significant differences in adaptive skills, with participants in the ASD/PSY group showing significantly lower scores. The impact of psychiatric comorbidities on the quality of life of individuals with ASD has already been demonstrated. In particular, the presence of co-occurring psychiatric disorders in ASD has been associated with greater perceived distress [75], worse quality of life [76,77,78], and greater deficits in social functioning [79]. In line with these studies, we found that children and adolescents with ASD and psychiatric comorbidities show a higher level of functional impairment than children with ASD without comorbidities. This helps to underscore the impact of co-occurring psychiatric disorders on the global functioning of youth with ASD and further highlights the pressing need for specific interventions on comorbidities. Mothers of participants in the ASD/PSY group exhibited greater stress than mothers of children with ASD alone. This confirms that the consequences of psychiatric comorbidities in youth with ASD go beyond individual impact. Numerous studies indicate that parents of children with neurodevelopmental disorder have higher stress levels than mothers of children without neurodevelopmental disorders [80,81,82]. Among neurodevelopmental disorders, parents of children and adolescents with ASD appear to be more stressed than parents of children with other kinds of neurodevelopmental disorders, such as intellectual disability and Down syndrome [83,84]. The results of the current study extend these previous findings, highlighting the crucial burden on parents of youth with ASD. Focusing on parental stress can have important clinical implications, as high levels of parental stress can influence parent–child interaction and child behavior. Therefore, the identification of psychiatric comorbidities in youth with ASD should also lead to timely and accurate assessment of parental stress levels.

The third aim of this study was to identify possible differences between preschoolers, school-aged children, and adolescents with a comorbid psychiatric disorder. Preschoolers exhibited lower IQ than adolescents, confirming that in preschoolers lower IQ could be a risk factor for the development of psychopathology [85]. We also found that preschoolers had worse adaptive skills than adolescents. This might suggest that psychiatric comorbidities particularly affect adaptive abilities in preschoolers, and this could be better explained considering that ADHD was the most frequently observed comorbidity in preschoolers. In fact, co-occurrence of ADHD symptoms in individuals with ASD is associated with worse adaptive functioning [86]. The results of the current study seem to suggest that, although psychiatric comorbidity overall leads to lower adaptive skills (as suggested by the comparison between ASD and ASD/PSY group), the association with ADHD results in the greatest functional impairment. Finally, preschoolers in the ASD/PSY group exhibited higher scores on the Restricted and Repetitive Behaviors domain of the ADOS-2 than adolescents. Again, the result could be explained, at least in part, considering that the vast majority of preschoolers in the ASD/PSY group have been diagnosed with ADHD. Indeed, previous research has indicated that children with ASD and co-occurring ADHD express greater severity of autistic symptoms [86,87]. In addition, symptoms of impulsivity and inattention have a strong phenotypic and genetic overlap with autistic traits, such as repetitive behaviors [88]. In a similar trend, Sokolova and collaborators [89] explored the relationship between ASD and ADHD symptoms by applying causal modeling. The authors identified a pathway between ASD and ADHD that links hyperactivity to repetitive behavior, suggesting that individuals who exhibit hyperactivity may have difficulty inhibiting motor behavior, and this difficulty may lead them to engage in motor behaviors that can be classified as stereotypic movements. However, further research is needed to understand the nature of the association between stereotypic movements and hyperactivity.

Some strengths and limitations should be considered when interpreting the results of the current study. The strengths of the present study include the large number of Italian children and adolescents with ASD, as well as the diagnosis of ASD established by a thorough clinical assessment according to DSM-5 criteria and supported by specific and widely used instruments, such as the ADI-R and ADOS, in addition to the assessment of each participant’s IQ and adaptive functioning, which is rare in studies with such large numbers. Limitations of the study include the cross-sectional research design. Longitudinal studies would be indicated to test how the prevalence of psychiatric comorbidities in ASD changes during development. Second, although we evaluated a large number of children and adolescents with ASD, it is important to recognize that the study was limited to patients attending the same Tertiary Care Hospital; therefore, we cannot exclude that by recruiting participants from community samples, and multiple sites, the results might change. Third, we focused on changes in the prevalence of psychiatric comorbidities in developmental age; therefore, further research is needed to clarify how the trajectories of psychiatric comorbidities also change in adults with ASD. In addition, sleep disorders were not considered in the present study, although they are highly frequent among individuals with ASD [12,90]. Fifth, we did not explore the association between psychiatric comorbidities and verbal ability, so their contribution to prevalence could not be analyzed. Moreover, females were underrepresented in our sample. Finally, considering that approximately 33% of the individuals with ASD have some form of intellectual disability [3], the choice to focus on high-functioning ASD may have excluded a substantial group of individuals with ASD. However, this choice allowed the exclusion of some clinical features, such as hyperkinesia or anxiety, which could be mainly related to the phenotype of intellectual disability [91]. Therefore, specific studies on the prevalence and distribution of psychiatric comorbidities in children with ASD and associated intellectual disability are necessary.

Despite these limitations, the present study provides critical new insights into the prevalence of psychiatric comorbid conditions in children and adolescents with ASD and specific features linked with these conditions. This study, indeed, is novel for several reasons. First, we provided evidence of the prevalence of comorbid psychiatric disorders in youth with ASD from a Mediterranean country; this is important since the prevalence variations reported in literature could be accounted to sociodemographic differences between populations [12]. Thus, contributions from underrepresented countries could significantly add to literature on this topic. Second, the current study deeply investigated functional impairment and ASD symptom severity associated with psychiatric comorbidities. Third, we investigated the impact of psychiatric conditions on parental stress, highlighting the importance of comprehensive caretaking. Finally, we characterized, across different age groups—including preschoolers—individual and family impacts of psychiatric comorbidities in youth with ASD.

## 5. Conclusions

The present study identified differences in the comorbidity of preschoolers, school-aged children, and adolescents with ASD, as well as specific profiles based on sex and associated psychiatric disorders. We also documented the impact of the presence of psychiatric comorbidities on the global functioning of children and adolescents with ASD, as well as maternal stress. Finally, we highlighted some peculiarities of individual and clinical features associated with psychiatric comorbidity in preschoolers with ASD compared with adolescents.

Future research should aim to fill research gaps by addressing multiple issues, such as exploring the prevalence and distribution of psychiatric comorbidities in ASD collecting data from underrepresented areas—e.g., low- and middle-income countries, and from large samples, including individuals with ASD and associated intellectual disability. Moreover, studies on the topic employing longitudinal designs are highly needed.

Early identification of ASD symptoms allows early individualized interventions to be initiated, reducing the risk of association with psychiatric comorbidities, such as anxiety and aggression [92]. Therefore, it is essential to conduct further research to characterize psychiatric conditions in children and adolescents with ASD to ensure adequate psychosocial and pharmacological care of comorbid disorders among people with ASD.

## Figures and Tables

**Table 1:** Distribution of comorbid psychiatric disorders among children and adolescents with ASD (%).

	Preschoolers (*n* = 221)	School-Age Children (*n* = 196)	Adolescents (*n* = 55)
ADHD	20.4	21	7.3
Anxiety/Obsessive-Compulsive Disorder	0.4	10.6	21.8
ODD	-	3.6	-
Mood Disorders	-	2	7.3
Tourette’s Disorder/Tic Disorder	-	2	5.4
Feeding and Eating Disorders	-	2	1.8
Other disorders	-	1	1.8

**Table 2:** Means and standard deviations for age, adaptive abilities, and ADOS-2 CSS scores. *: *p* < 0.05.

	ASD	ASD/PSY	Post-Hoc Comparisons (Tukey HSD Test) *p*-Values
Age	6.66 ± 3.24	8.25 ± 3.47	<0.001 *
Adaptive skills	67.32 ± 18.05	63.1 ± 15.37	0.02 *
ADOS-CSS Social Affect	5.69 ± 1.52	5.98 ± 1.65	0.07
ADOS-CSS Restricted and Repetitive Behaviors	6.81 ± 1.62	6.61 ± 1.98	0.26
ADOS-CSS Total score	5.75 ± 1.4	5.85 ± 1.45	0.51
PSI Total score	76.78 ± 22.43	83.23 ± 22.51	<0.01 *

## Data Availability

The data presented in this study are available on request from the corresponding author.
